# Tempo-spatial variations of the Ryukyu Current southeast of Miyakojima Island determined from mooring observations

**DOI:** 10.1038/s41598-020-63836-5

**Published:** 2020-04-20

**Authors:** Ruixiang Zhao, Hirohiko Nakamura, Xiao-Hua Zhu, Jae-Hun Park, Ayako Nishina, Chuanzheng Zhang, Hanna Na, Chanhyung Jeon, Ze-Nan Zhu, Hong Sik Min

**Affiliations:** 1 State Key Laboratory of Satellite Ocean Environment Dynamics, Second Institute of Oceanography, Ministry of Natural Resources, Hangzhou, 310012 China; 2 Southern Laboratory of Ocean Science and Engineering (Guangdong, Zhuhai), Zhuhai, 18 China; 3 Faculty of Fisheries, Kagoshima University, 4-50-20, Shimoarata, Kagoshima, 890-0056 Japan; 4 School of Oceanography, Shanghai Jiao Tong University, Shanghai, 200030 China; 5 Department of Ocean Sciences, Inha University, Incheon, Korea; 6 School of Earth and Environmental Sciences, Seoul National University, Seoul, 08826 Korea; 7 Department of Marine Science and Biological Engineering, Inha University, Incheon, Korea; 8 Ocean Circulation and Climate Research Center, Korea Institute of Ocean Science and Technology, Busan, Korea

**Keywords:** Physical oceanography, Physical oceanography

## Abstract

The origin, structure, and variability of the Ryukyu Current (RC) have long been debated, mostly due to limited observations. A mooring array, deployed for two years southeast of Miyakojima in the southern portion of the Ryukyu Island chain, has provided, for the first time, data confirming the existence and revealing the characteristics of the RC in that upstream region, including its velocity structure and variability. The observations show a shoreward-intensified current flowing northeastward, with a subsurface core located near the 1,000 m isobath and having a record-long mean speed of up to 19.4 cm s^−1^ at 500 m depth. Estimated volume transport across the observation section had mean 9.0 Sv (1 Sv = 10^6^ m^3^ s^−1^) and standard deviation 8.7 Sv. The RC shows significant barotropic character compared with other similar mid-latitude currents.

## Introduction

**Figure 1:**
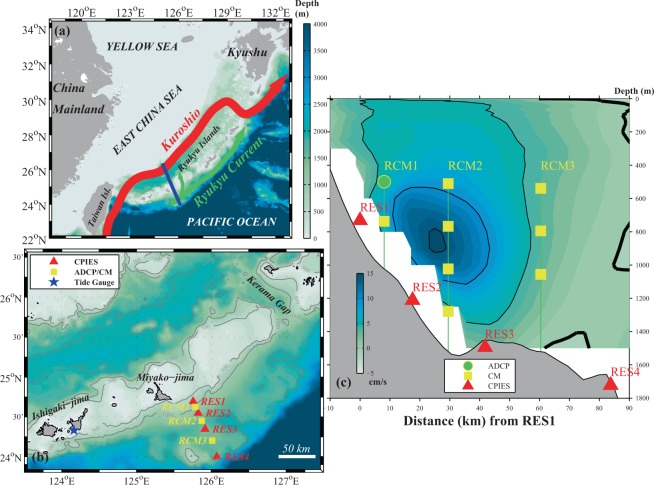
(**a**) Map of the East China Sea and northwestern Pacific Ocean. Kuroshio Current, Ryukyu Current (RC), and mooring section are indicated by red, green, and blue lines, respectively. (**b**) Mooring locations offshore of Miyakojima Island. Tall current meter (CM)/acoustic Doppler current profiler (ADCP) moorings (RCM1, RCM2, RCM3) are indicated by yellow squares; current and pressure-recording inverted echo sounder (CPIES/PIES) moorings are indicated by red triangles; the Ishigaki tide-gauge station is indicated by a blue star. (**c**) Cross-section of the mooring array that observed the RC. Background colour shows the long-term (Jan. 2000 to Jun. 2017) mean velocity (cm s^-1^) across the section, with black contours at intervals of 5 cm s^−1^, from the HYbrid Coordinate Ocean Model reanalysis. Bold black contour lines indicate zero velocity.

The Kuroshio is the strongest western boundary current in the Pacific Ocean. Consequently it has long been the focus of many scientific studies^1,2^. After it enters the East China Sea (ECS), the Kuroshio flows northeastward, west of the Ryukyu Island chain, until it reaches the region south of Kyushu, Japan and exits through the Tokara Strait (Fig. 1a). However, studies^3,4^ have also indicated the existence of another strong portion of the western boundary current, namely the Ryukyu Current (RC) flowing northeastward just east of the Ryukyu Island chain, accounting for the large difference in volume transport of the Kuroshio between the ECS (19 − 28 Sv; 1 Sv = 10^6^ m^3^ s^−1^)^5–7^ and the sea off southern Japan (42~65 Sv)^8,9^. Compared to the Kuroshio west of the Ryukyu Island chain, which is surface intensified and relatively stable, the RC features a strong subsurface velocity core^10,11^ and large spatio-temporal variability caused by mesoscale eddies propagating from the Pacific^12,13^. Previous studies have also suggested the RC’s variability may be related to that of the Kuroshio East of Taiwan^14–16^. Interactions between the RC and the Kuroshio via the Kerama Gap (Fig. 1b) contribute to the variability of the Kuroshio in the ECS and play a key role in water mass exchange between the ECS and the North Pacific^17–20^. The RC carries a large mass of water, heat, and nutrients poleward^21,22^, greatly enhancing the Kuroshio when the two currents merge east of the Tokara Strait.

Although the RC is important to the circulation of the North Pacific, its mean structure and spatio-temporal variability remain unclear, due to limited observations. Snapshots of hydrographic casts^23,24^ can be greatly affected by mesoscale eddies; therefore, long-term mooring deployments are needed to remove aliases. Previous mooring observations have revealed the velocity structure and variability of the RC southeast of Amami-Oshima^3^ and Okinawa^21,25^, which are in the downstream region of the RC. In the upstream region, two moored current meters showed a persistent northeastward current southeast of Miyakojima^26^ but were not able to determine the structure of the RC there.

Numerical models such as those of Thoppil *et al*.^26^, You & Yoon^27^, and Nakamura *et al*.^28^ are helpful for filling in spatial and temporal gaps in observations and revealing the physical mechanisms of the generation and evolution of the RC. Among these models, the 1/12° HYbrid Coordinate Ocean Model (HYCOM) reanalysis was reported to perform the best, in terms of its consistency with previous observations in the RC region^3,29^. However, the subsurface intensified velocity core in the upstream region of the RC has never before been validated with observations. Determining the velocity field in this region is key to clarifying the mechanism of formation of the RC, which would lead to a better understanding of the water mass budget in the northwestern Pacific and of the interactions between the RC and the Kuroshio.

In this paper, we present the structure of the current southeast of Miyakojima Island as observed for 2 years by a mooring array. This array consisted of multiple current-sensing instruments (up to 13 instruments of 3 types on 7 moorings, *see next section*) positioned southeast of Miyakojima, beneath a satellite altimeter track. Using these current measurements, we investigate the mean and time varying structure and volume transport (VT) of the RC in the southern Ryukyu region, as well as the influence of mesoscale eddies entering from the east, and we compare our results with model results from HYCOM.

## Results

This section shows the current observations and derived estimates from the mooring array. The mooring array (Fig. 1c) was comprised of four current-and-pressure-recording inverted echo sounders (CPIESs), three tall moorings, each with multiple current meters (CMs), and one acoustic Doppler current profiler (ADCP). The CPIES is a bottom-moored instrument which records bottom pressure and acoustic travel time from the seafloor to the surface and back; in addition, it incorporates a CM-type sensor and records the current velocity (speed and direction) 50 m above the seafloor. The CMs provided point measurements of current velocity at each instrument depth. The ADCP (at the top of one of the tall moorings) measured upper-layer currents from a nominal depth of 580 m to the surface.

**Figure 2:**
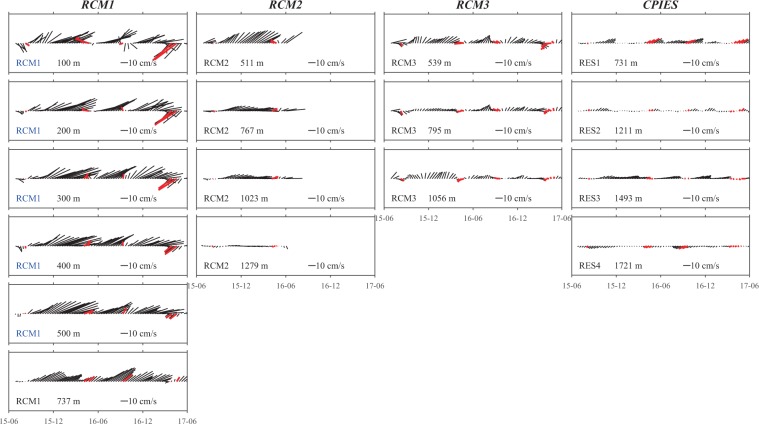
Time series vector plots for all current-measurement records. Plotted vectors represent 10-day vector-averages of the measured currents. Nominal depths of the measurements are shown at the lower-left of each box. Note that the 10 cm/s scale arrow in each panel points due east. Velocities measured by the ADCP on RCM1 are shown at 100 m depth intervals. Red vectors indicate times when VT was below its mean minus one standard deviation. Tick marks on the horizontal axes are at half-year intervals.

**Figure 3:**
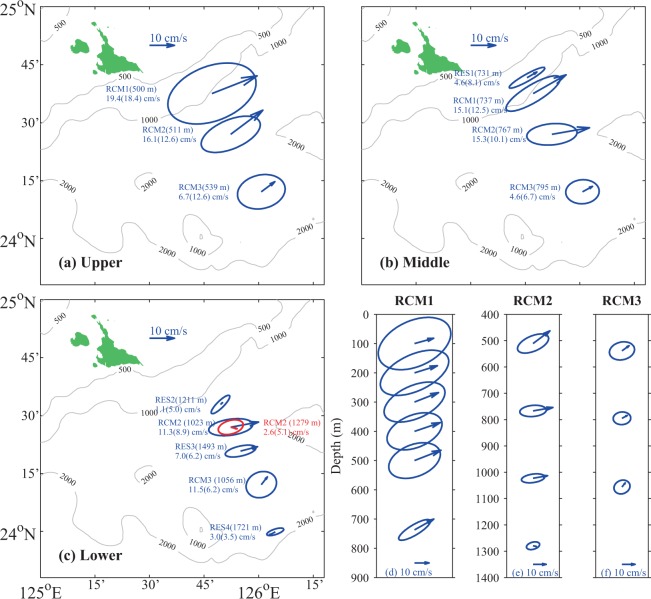
Vector-average velocities (arrows) and standard deviation ellipses for measured currents shown for three depth layers (**a–c**) and for three tall-mooring sites (**d–f**). Station name (depth) and current speed (ellipse semi-major axis) as marked. Bathymetric contours are in meters. Scale arrow (10 cm/s) in each panel also indicates eastward direction.

Here we first show the observations from all the current-measurement results (Figs. 2, 3). Time series of measured velocity vectors (Fig. 2) show temporal variations of the currents, which are critical in determining whether the RC is persistent or intermittent. Notably, the velocity vectors tend to be persistently toward the northeast for the shoreward stations (i.e., RES1, RCM1, RES2, RCM2, and RES3), with one significant exception: under the influence of a strong cyclonic eddy in April 2017, the upper layer at RCM1 shows reverse velocity vectors. Nevertheless, statistics of velocities in the vicinity of the RC core (hereafter defined as the location of the maximum current speed, about 500 m depth at the RCM1 mooring) support the conclusion of a mean northeastward flow: for the eastward and northward velocity components the 99% confidence intervals are 17.9 ± 1.7 cm/s and 7.2 ± 1.2 cm/s, respectively. VT across the section was positive during 618 days (87% of 713 days total). Thus, we can conclude that although the RC can be intermittent under the influence of strong cyclonic eddies, it appeared as a strong northeastward current during most periods.

In order to illustrate better the velocity structure, Fig. 3 shows mean velocity vectors and standard deviation ellipses from the current measurements. To simplify interpretation, the moored instruments are grouped by depth: the upper layer is defined as shallower than 600 m; the middle layer is 600–900 m; the lower layer is deeper than 900 m. In the upper layer (Fig. 3a), the direction of all the mean velocity vectors is northeastward and their strength intensifies shoreward; the maximum, recorded at the RCM1 mooring, reached 19.4 cm s^−1^. In the middle layer (Fig. 3b), the mean velocity vectors are again oriented toward the northeast, although slightly weaker than those of the upper layer. RES1 showed a weaker current than RCM1, thus indicating that the RC core was seaward of RES1. In the lower layer (Fig. 3c), the mean direction of all currents was northeastward except in the countercurrent that occurred at RES4. Velocities decreased sharply at depths below 1,000 m: RCM2 mean speed at 1,023 m was 11.3 cm s^−1^, while at 1,279 m it reached only 2.6 cm s^−1^. RES2 also recorded a weak northeastward current with a mean speed that reached only 1.1 cm/s at a depth of 1,211 m. This indicates that there is a nearly motionless layer below the RC core, with strong velocity shear up to 10 cm s^−1^ within 200 m of the bottom. It was interesting that the CPIES station seaward of RCM2 (RES3) recorded a strong northeastward current with a mean speed of 7.0 cm s^−1^; this was much faster than that recorded by RCM2 at 1,279 m (2.6 cm s^−1^), though RES3 was more than 200 m deeper (1,493 m).

Figure 3d–f respectively show the average vertical structure of the velocities at the RCM1, RCM2, and RCM3 tall moorings. The current profile at RCM1 showed weak baroclinic characteristics, with only a small and gradual increase in mean velocities from the surface to 500 m, and a weak decrease at 737 m, while variability of the currents shown by the standard deviation ellipses decreased monotonically with depth. The shallowest current meters at RCM2 and RCM3 were at 511 and 539 m depth, so only current profiles deeper than the depth of the RC core were recorded there. Each of these showed baroclinic characteristics: both mean velocities and variabilities decreased with depth. It is important to note that RCM2 recorded a strong (8.7 cm s^−1^) velocity shear between 1023 m and 1279 m in the near-bottom layer of the RC. Mean velocities at RCM3 were smaller than those at both RCM1 and RCM2 and the deviation ellipses at RCM3 were more nearly circular, which implies that the RC is mainly confined shoreward of RCM3.

**Figure 4:**
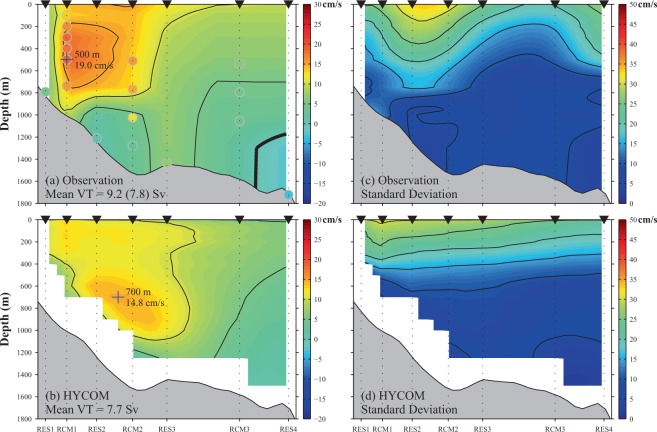
Temporal mean (left panels; a&b) and standard deviation (right panels; c&d) of velocity across a section of the Ryukyu Current during the observational period (June 2015 to June 2017) based on observations (upper panels; a&c) and HYbrid Coordinate Ocean Model (HYCOM) outputs (lower panels; b&d). Contour interval is 5 cm/s and the bold black line indicates a value of zero. Small coloured circles in (**a**) show mean velocities from direct observations. The position of maximum velocity of the current core is indicated with a cross. Mean volume transports (VTs) are given in the lower left-hand corners; in (**a**), mean VT for the same averaging area as (**b**) is given in parentheses.

To obtain further information on the velocity field across the section, we used data from the four CPIESs to provide estimates and fill in the “gaps” that were not measured by the ADCP and current meters. The resulting time-averaged cross-section velocity is presented in Fig. 4a. The estimates closely resemble the velocities recorded at the observational positions of the moorings (small coloured circles in Fig. 4a) because of the weighed estimation method used (see Supplementary Information). The differences in RCM2 are largely caused by the fact that RCM2 was lost in the second year of observations; therefore, only the first-year means are presented. A strong RC subsurface core is identified at depths between 200 and 750 m near the continental shelf break (RCM1) with mean speeds exceeding 15.0 cm s^−1^. The maximum (blue cross in Fig. 4a) reached 19.0 cm s^−1^ at 500 m for the RCM1 mooring. The core of the RC was greatly constrained by the continental shelf break shown at the 1,000 m isobath. Currents below 1,000 m were very weak; this depth clearly delineated the lower edge of the RC core. Seaward of RES3, the northeastward currents became much weaker and a countercurrent was identified, at depths greater than 1,200 m, near RES4. The temporal mean volume transport across the section was calculated as 9.0 Sv. The standard deviation section (Fig. 4c) showed large deviations at the surface layer near RES2 and RES4, which may be explained by great variabilities caused by eddies: the larger deviation near RES4 was likely caused by direct eddy impacts because it is located the most seaward; while the larger deviation near RES2 may be explained by alongstream propagation of eddy signals. At the RC’s core, the standard deviation reached only ~9 cm/s, much smaller than its temporal mean (19.0 cm/s). Thus, the RC was relatively stable.

## Discussion

Because *in situ* observations are scarce in the RC region, reanalysis data are used as a reference to outline the basic structure and variability of the RC system. The 1/12° global HYCOM reanalysis has been reported to perform well^26,29^ in simulating the RC system when compared to previous long-term tall-mooring observations, but its ability to reproduce RC subsurface structure in the upstream region has never before been validated with *in situ* measurements. Our mooring observations offer a unique opportunity to do this. For comparison with Fig. 4a,b shows the temporal mean of the velocity structure across the section from the HYCOM outputs, averaged during our observational period. The reanalysis results reproduced certain features that match our observational results: the shear under the velocity core is large so the near-bottom flows are weak, and the northeastward currents become gradually weaker seaward of RES3. The temporal mean volume transport (VT) from the HYCOM outputs, was calculated as 7.7 Sv. Considering that the topography of the model is smoothed, not realistic, we recalculated the estimated VT in Fig. 4a by dropping the data points that were masked in the model. The new estimated mean VT, 7.8 Sv, is close to that from HYCOM.

However, there were several important differences between our observations and the HYCOM outputs: for example, the simulated RC core was located near RCM2, with a maximum velocity of 14.8 cm s^−1^ at 700 m. Compared to our observations, the simulated RC core was slightly seaward, deeper, and weaker. We attribute the differences to a systematic bias in the model because similar problems were also reported in previous comparisons in the downstream region^27^. The deeper simulated RC core might lead to an underestimation of its heat transport as temperature is higher in shallower water. We note that the RC core’s tilting along the topography for HYCOM differed from the observed non-slanted, nearly-barotropic structure. This might have been caused by the choice of lateral viscosity in the model. It has been reported^28^ that large lateral viscosities can dampen higher-mode baroclinic Rossby waves, leading to tilted RC structures. Sensitivity experiments showed tilted structures diminishing after reducing the lateral viscosity coefficient. The deep countercurrent observed in RES4 was not present in the simulation, probably due to limitations in the model’s topography. Nevertheless, the currents simulated at the RES4 location were also quite weak and the countercurrent was present in the model products from previous years (Fig. 1c). These comparisons may be helpful in eliminating biases and improving the performance of the model.

**Figure 5:**
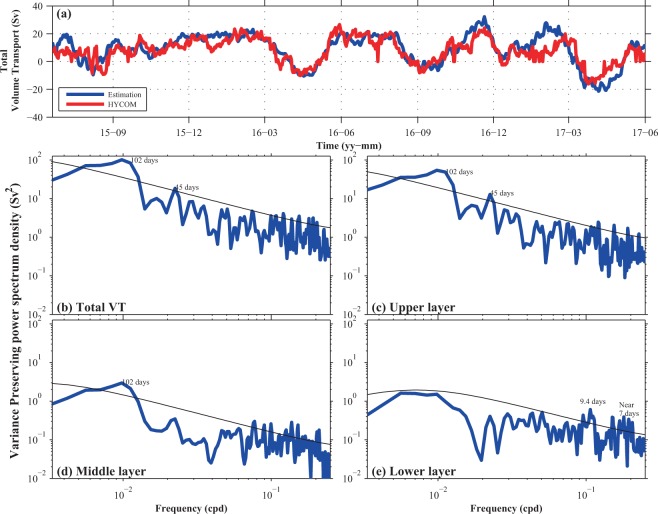
(**a**) Time series of the total volume transport (VT) according to our estimate (blue line) and from the HYbrid Coordinate Ocean Model (HYCOM) (red line). The power spectra of (**b**) the total VT, (**c**) upper-layer VT, (**d**) middle-layer VT, and (**e**) lower-layer VT are shown below. Black lines indicate 95% confidence levels.

The RC system was reported to be greatly influenced by frequent mesoscale eddies. We present the time series of the total VT from our estimates and from the HYCOM outputs, in Fig. 5a. The two VT time series are similar with characteristic periodicities of nearly three months, which is about the interval between arrivals of Pacific mesoscale eddies^12,13^. It should be noted that velocities near the RC core were always northeastward (Fig. 2), so the negative VT, which was caused by a southwestward flow in the eastern part of the section, does not mean the RC was intermittent or absent. Low-frequency signals were not significant in the lower layer; instead, high-frequency signals peaking at periods around 7 and 9 days prevailed.

**Figure 6:**
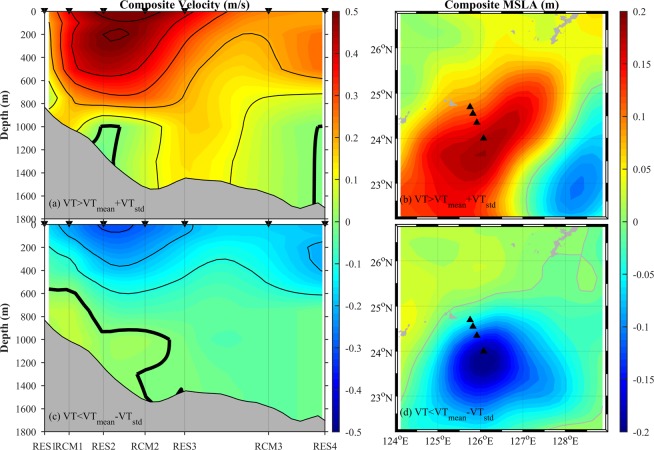
Left panels: composite distribution of mean velocity across the section when the VT is (**a**) above its mean plus one standard deviation (VT_mean_ + VT_std_) and (**c**) below VT_mean_ - VT_std_; the contour interval is 0.1 m/s and bold black lines indicate values of zero. Right panels: composite distribution of merged sea level anomaly (MSLA) when the VT is (**a**) above VT_mean_ + VT_std_ and (**c**) below VT_mean_ - VT_std_. Small black triangles indicate mooring positions.

To investigate the time-varying velocity structure, we conducted composite analysis (Fig. 6) when the VT was above/below its mean plus/minus one standard deviation (VT_mean_ ± VT_std_). When the VT was above VT_mean_ + VT_std_ (Fig. 6a), the maximum positive velocity (defined as the composite RC core, distinguished from the mean RC core in the previous section) appears at the surface near RES2, with a mean speed reaching 55 cm s^−1^. The appearance of surface maximum structure should not be interpreted as lifting of the whole RC, because eddies strongly impact the surface, masking the RC’s ‘true’ structure. The location of the composite RC core is 10 km further seaward than the mean RC core. A countercurrent emerges near the bottom at RES2 and RES4. Composite merged sea level anomaly (MSLA, from Copernicus) shows (Fig. 6b) that the observational sites are under the influence of a strong anticyclonic eddy, appearing as an ellipse elongated along the local isobath. The irregularity arises from the fact that the composite averages the MSLA of anticyclonic eddies propagating from different directions.

When the VT is below VT_mean_ − VT_std_ (Fig. 6c), the direction of currents in the upper layer above 600 m turns southwestward. However, currents within about 300 m of the bottom shoreward of RES3 still flow northeastward. The composite RC’s core is located between RES1 and RCM1 near 1,000 m, with a maximum mean speed reaching only 6 cm s^−1^. It seems that the composite RC flows deeper than the mean RC, so current at the bottom of RES2 changes its direction and flows northeastward. At the same time, currents near the bottom of RES4 still flow southwestward, suggesting little variability there. Composite MSLA (Fig. 6d) shows that the observational sites were under the influence of a strong cyclonic eddy. From this analysis, we deduce that eddies can significantly change the velocity structure of the RC.

Because the VT estimated above was greatly affected by mesoscale eddies, further examination of long-term variations and statistical characteristics of the RC requires longer-term estimates of VT time series. To achieve this, we used sea surface height difference (SSHD) across the RC near our section as a proxy. First, we calculated the SSHD between RES4 and RES1 from the Copernicus altimeter products. We then applied a 10-day running average, obtaining a time series SSHD_sat-sat_. The correlation (*r* = 0.70, which exceeds the 99% confidence level) between the observations and SSHD_sat-sat_ was not as strong as the near 0.90 values found in previous studies in other regions such as the Kuroshio^9,16^, the RC near Okinawa^30^, and the South China Sea^31,32^, because high-frequency processes were strong near RES1 (which was also seen in CPIES bottom pressure records) and may contaminate the altimeter data through aliasing. Therefore, we used the sea-level difference between RES4 and Ishigakijima instead (see Fig. 1b). The Ishigaki tide-gauge data from the Japan Oceanographic Data Center (JODC), consists of tidal height measured every hour since 1969. The SSHD_sat-TG_ between RES4 and Ishigakijima has a correlation coefficient of 0.87 with the VT when applied with a 10-day running average. By employing a linear correlation between the SSHD_sat-TG_ and the estimated VT during the observational period, the long-term mean VT was estimated as 7.2 Sv.

**Figure 7:**
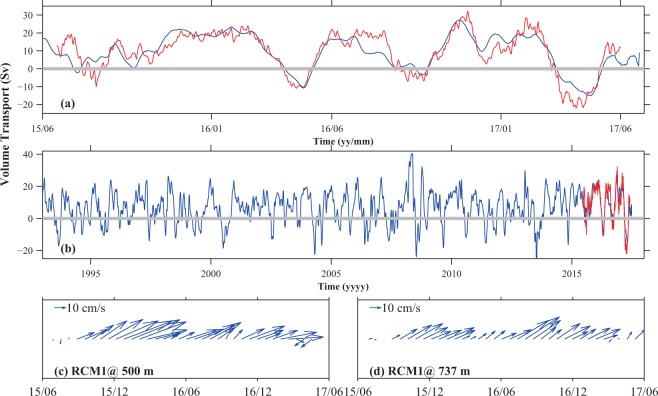
(**a,b**) VT time series estimated from mooring data (red line) and from SSH difference (blue line) between RES4 and Ishigakijima during the observation period (**a**) and since 1993 (**b**). **(c,d)** Velocity vectors from current measurements near the RC core.

The long-term VT rules out the possibility that the northeastward RC during the observational period is just the result of frequent eddy impingement: we noted that in April 2017, just before the June recovery cruise, this section experienced strong impacts of a cyclonic eddy, and the long-term VT (Fig. 7b) achieved nearly its lowest value over the 25-year period, but our current measurements near the RC core turned southwestward only weakly (Fig. 7c). We conclude that the RC exists southeast of Miyakojima Island. The long-term VT is also important to our understanding of the volume transport budget here: the difference in VT (2.7 Sv) between that at Miyakojima Island (7.2 Sv) and VT southeast of Okinawa Island (4.5 Sv) is likely accounted for by VT through the Kerama Gap (mean 2.0 ± 0.9 Sv during 2009–11, from the northwestern Pacific into the East China Sea^19^). Thus we may regard the currents through the Kerama Gap as a branch of the RC intruding into the East China Sea.

**Figure 8:**
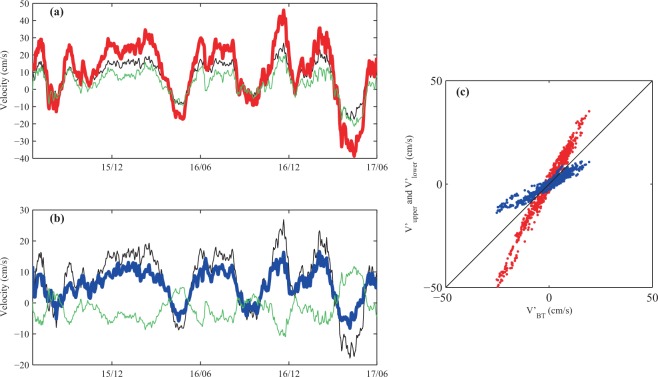
(**a**) Time series of the velocity components in the upper layer: v_upper_ (red), v_BTupper_ (black), and V_BCupper_ (green). **(b)** Time series of the velocity components in the lower layer: v_lower_ (blue), v_BTlower_ (black), and V_BClower_ (green). **(c)** Upper ocean (v’_upper_, red) and deep-ocean (v’_lower_, blue) velocity anomalies as functions of the barotropic velocity anomalies. Black line shows 1:1 ratio.

From observations reported in the previous section, we see that the temporal-mean RC contains a significant barotropic component. Here, the barotropic and baroclinic components were quantified using the method documented in ref. ^17^. The interface was set to be 500 m. According to the results, the baroclinic (BC) component is weaker than the barotropic (BT) component in both the upper layer and the lower layer (Fig. 8a,b). Thus the RC is predominantly a barotropic current, which is interesting because currents at these latitudes tend to be mainly baroclinic: for example, the baroclinic component of the Kuroshio is much larger than its barotropic component in the upper layer, as shown in ref. ^17^. Nevertheless, in terms of variability, the baroclinic component dominates the upper-layer variation (Fig. 8c, red dots fall on a v’_upper_:v’_BT_ line steeper than 1:1), whereas the barotropic component dominates the lower-layer variation (Fig. 8c, blue dots fall on a v’_lower_:v’_BT_ line less steep than 1:1), which resembles the Kuroshio case^16^. Thus, the RC is mainly a barotropic current in the temporal-mean sense, but with strong baroclinic variability.

The hypotheses for the formation mechanism of the Ryukyu Current can be characterized as follows: a branch of the Kuroshio east of Taiwan^23^; enforced by the anticyclonic recirculation southeast of Okinawa Island^27^; and the effect of baroclinic-mode topographic Rossby waves on the subsurface current-core formation^28^. Because the observed Ryukyu Current was quite persistent despite impacts from strong cyclonic eddies, it is unlikely that the Ryukyu Current was solely eddy-induced. Moreover, the mean flow is approximately barotropic removing the eddies’ baroclinic velocity structure, suggesting other candidates for the RC’s formation mechanism. Based on the analysis of HYCOM^29^, a branch of the Kuroshio east of Taiwan with consistent velocities in the vertical, feeds the RC and may explain the barotropic feature of the RC. And the eddies contribute to its mean VT and variability along the streamline, which is consistent with previous studies upstream^15,16^. We had no observational evidence to prove or deny that the subsurface current core is caused by baroclinic-mode topographic Rossby waves. Therefore, the formation mechanism of the subsurface current core is still uncertain in this study. Further studies from observational data analyses and numerical experiments are needed in the future to clarify the formation mechanism of the RC.

From the mooring observations, we determined the existence of the Ryukyu Current in an upstream region southeast of Miyakojima Island and demonstrated its intensified subsurface velocity structure and VT variability for the first time. Further long-term observations are needed in this region of the RC to delineate its detailed velocity structure and its relationship with the RC downstream. Interactions between the RC and the Kuroshio Current would also be an interesting subject for future study.

## Methods

From June 2015 to June 2017, an international cooperative research project called the “Joint Kuroshio and Ryukyu Current System Study” (JKRYCSS) was conducted by scientists from Japan, China, and South Korea. Tall CMs, ADCPs, and CPIESs were deployed on either side of Miyakojima Island to observe the Kuroshio Current and the RC simultaneously. These mooring sites lay along the altimeter track used by TOPEX/Poseidon and Jason-1/2/3, which is roughly perpendicular to the isobath of the continental shelf break. On the RC side, the moorings were intended to provide observations of the RC core that had been simulated by HYCOM (Fig. 1c). In 2016, we recovered the three tall CM moorings to retrieve their data and replace their batteries. The recovered instruments were deployed again for the second year of observation. Unfortunately, RCM2 was lost after re-deployment so only its first-year records were obtained. Complete 2-year records were successfully retrieved from all the other instruments.

Attached to the top of mooring RCM1 was an upward-looking 75 kHz RDI Workhorse ADCP at a nominal depth of 580 m. The ADCP was set to measure the velocity profile of the upper layer twice every hour. Its measurement data were interpolated to a vertical grid with 10 m intervals at depths of 50 m to 500 m. With a current meter at 737 m depth, the observational range of RCM1 covered almost the entire water column. Each CPIES measured the acoustic round-trip travel time (τ) and variations in bottom pressure, while the attached Aanderaa current meter measured current velocity 50 m above the seafloor. The quality of all CPIES data was controlled following standard methods^33,34^.

To determine the thermohaline vertical structure and its variation, we constructed gravest empirical modes (GEM)^35^ by collecting historical conductivity, temperature, depth (CTD) and Argo profiles southeast of Miyakojima Island. A GEM is essentially a lookup table which enables the conversion of acoustic-travel-time data (τ) into temperature or specific-volume-anomaly profiles. The optimal interpolation method^35,36^ was used to estimate the temperature and salinity distribution of the section at a spatial resolution of 2 km. The correlation length scale for the τ values was empirically determined to be 122 km. The baroclinic shear of the velocity across the section was calculated using the thermal-wind relationship by assuming that the current was geostrophic. Mean values of the altimeter-derived surface geostrophic current and tall mooring observations were used to determine the offsets of the leveled τ values (please refer to section 2.1 in Supplementary Information). To improve the accuracy of estimated velocities, we combined the baroclinic velocity shear estimated using the CPIES data with all the direct current observations (from the ADCP and CMs on the tall moorings, and CMs on the CPIESs). Various reference levels and depth-dependent weights were adopted to make the estimates using this process. This method ensures that all the current measurements were used optimally in the estimation process. Details for these procedures are given in the Supplementary Information section.

In addition to the mooring observation records, the 1/12° HYCOM reanalysis data were used for comparison. This product assimilates observations such as sea surface height (SSH) and sea surface temperature (SST) taken from satellite measurements with *in situ* measurements such as temperature and salinity profiles from CTD profilers, expendable bathythermographs, gliders, and Argo casts^3^.

## Supplementary information

Supplementary Information
