# Supplementary material for: Tempo-spatial variations of the Ryukyu Current southeast of Miyakojima Island determined from mooring observations
Source: Sci Rep. 2020 Apr 20;10:6656. doi: 10.1038/s41598-020-63836-5 (PMC7170915; doi:10.1038/s41598-020-63836-5)
Supplement: Supplementary file 1 — Supplementary Information [file 41598_2020_63836_MOESM1_ESM.docx]

Supporting Information for

**Tempo-spatial variations of the Ryukyu Current southeast of Miyakojima Island determined from mooring observations**

# Ruixiang Zhao^1,2^, Hirohiko Nakamura^3^, Xiao-Hua Zhu^1,2,4,*^, Jae-Hun Park^5^, Ayako Nishina^3^, Chuanzheng Zhang^1,2^, Hanna Na^6^, Chanhyung Jeon^7^, Ze-Nan Zhu^1^, and Hong Sik Min^8^

*Corresponding author: [xhzhu@sio.org.cn](mailto:xhzhu@sio.org.cn)

^1^State Key Laboratory of Satellite Ocean Environment Dynamics, Second Institute of Oceanography, Ministry of Natural Resources, Hangzhou, 310012, China

^2^Southern Laboratory of Ocean Science and Engineering (Guangdong, Zhuhai), Zhuhai,18 China

^3^Faculty of Fisheries, Kagoshima University, 4-50-20, Shimoarata, Kagoshima, 890 0056, Japan

^4^School of Oceanography, Shanghai Jiao Tong University, Shanghai 200030, China

^5^Department of Ocean Sciences, Inha University, Incheon, Korea

^6^School of Earth and Environmental Sciences, Seoul National University, Seoul 08826, Korea

^7^Department of Marine Science and Biological Engineering, Inha University, Incheon, Korea

^8^Ocean Circulation and Climate Research Center, Korea Institute of Ocean Science and Technology, Busan, Korea

The mooring system is comprised of several CMs, CPIESs, and an ADCP located at different sites and depths (Table S1). The CPIESs alone could estimate the geostrophic velocity distribution across the section. However, because the velocity field of the Ryukyu Current (RC) is quite complex, features such as the subsurface core and the large velocity shear below it are quite difficult to reproduce using CPIESs; the velocity shear was recorded by the CMs. To improve our estimate of the velocity structure and obtain a more accurate volume transport (VT) time series, all the direct current measurements were utilized to create the reference velocities using the following procedures.

**Table S1. Data on the moorings**

| Station | Instrument | Latitude (N) | Longitude (E) | Observation  Period | Nominal  Depth (m) |
| --- | --- | --- | --- | --- | --- |
| RCM1 | ADCP (upward-looking) | 24^o^37.56’ | 125^o^47.16’ | 2015/06-  2017/06 | 580 |
|  | CM |  |  |  | 737 |
| RCM2 | CM | 24^o^27.00’ | 125^o^52.32’ | 2015/06-  2016/06 | 511 |
|  | CM |  |  |  | 767 |
|  | CM |  |  |  | 1023 |
|  | CM |  |  |  | 1279 |
| RCM3 | CM | 24^o^12.12’ | 126^o^0.72’ | 2015/06-  2017/06 | 539 |
|  | CM |  |  |  | 795 |
|  | CM |  |  |  | 1056 |
| RES1 | CPIES | 24^o^41.64’ | 125^o^45.48’ | 2015/06-  2017/06 | 732 |
| RES2 | CPIES | 24^o^32.88’ | 125^o^49.50’ | 2015/06-  2017/06 | 1212 |
| RES3 | CPIES | 24^o^20.82’ | 125^o^55.02’ | 2015/06-  2017/06 | 1494 |
| RES4 | CPIES | 23^o^59.88’ | 126^o^4.56’ | 2015/06-  2017/06 | 1721 |

**1. Baroclinic velocity estimation**

This section describes the procedures for estimating the baroclinic velocity estimation from CPIES observations.

**1.1 Gravest Empirical Mode construction**

To estimate the structure and variation of the thermohaline from CPIES observations, we constructed a GEM (Watts et al., 2001; Donohue et al., 2010) by collecting historical temperature and salinity profiles from 1362 historical conductivity, temperature, and depth (CTD) measurements and 419 Argo casts in the region of the RC (Fig. S1a). CTD data were taken from the Japan Meteorological Agency (JMA) and the Kagoshima-maru vessel (Fig. S1b). Argo data were downloaded from the China Argo Real-time Data Center (http://www.argo.gov.cn/argo-eng/index.asp). A GEM is essentially a look-up table that interprets leveled round-trip travel times ($\tau_{ref}$) to temperature (Fig. S1c) or specific volume anomaly profiles (Fig. S1d). CTD and Argo data were collected in a large area to improve the GEM accuracy and increase the range of $\tau_{ref}$ for estimation. The mean GEM error for temperature (specific volume anomaly) is 0.97 °C (2.78 × 10^-7^ m^3^ kg^-1^) for the upper 300 m and 0.31 °C (6.81 × 10^-8^ m^3^ kg^-1^) for the full water column (Fig. S1e and Fig. S1f, respectively).


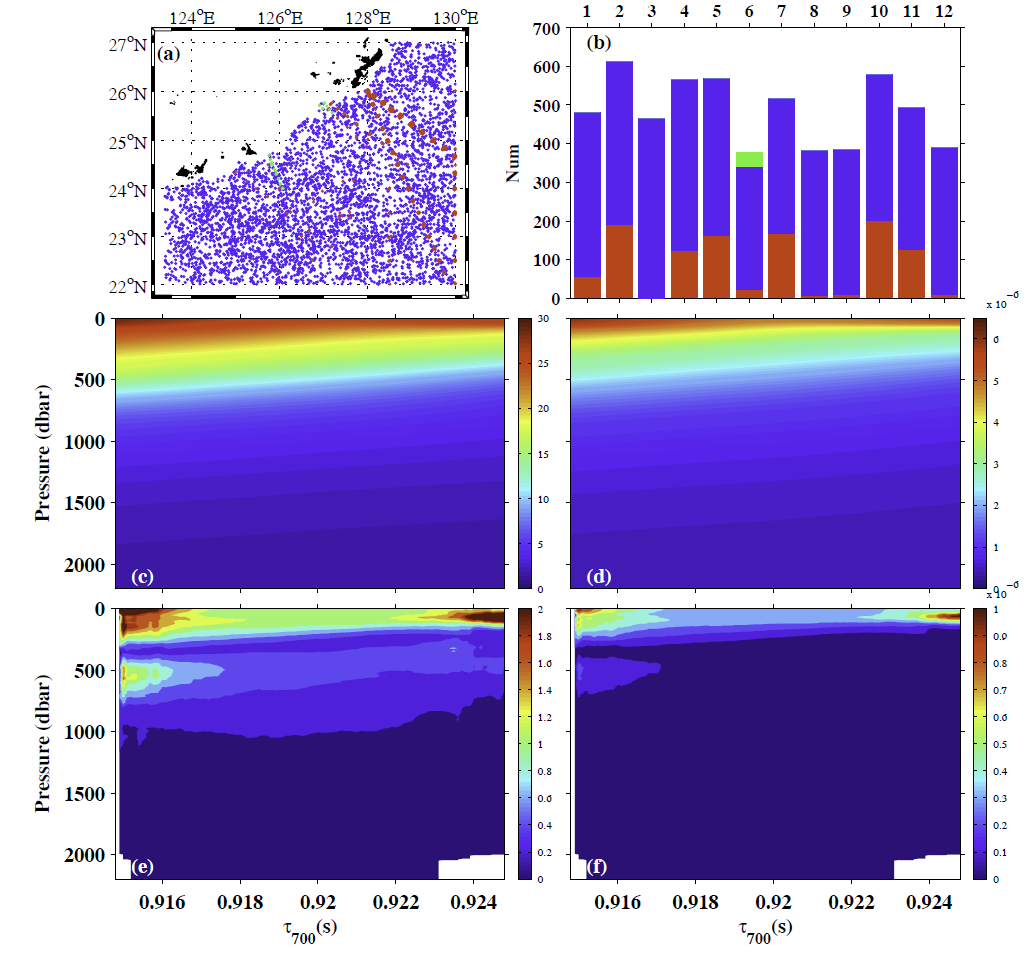


**Figure S1.** (a) Geographic distribution of conductivity, temperature, and depth (CTD) profiles (red dots represent Japan Meteorological Agency (JMA) data; green dots represent data from the Kagoshima-maru) and Argo casts (blue dots are data from the China Argo center) used to construct the gravest empirical mode (GEM) . (b) Monthly quantitative distribution of CTD profiles (red bars are from JMA data; green bars from the Kagoshima-maru) and Argo casts. The GEMs for (c) temperature and (d) specific volume anomaly. (e) and (f) show the same data (c) and (d) with the errors included.

**1.2 Travel time (**$\boldsymbol{\tau}$**) correction**

The observed values of $\tau$ were leveled to a reference depth of 700 *m*. As this procedure can introduce errors that lead to systematic biases, offsets of the leveled$\tau$ ($\tau_{700}$) needed to be determined. However, as the depth of RES1 was close to 700 *m*, its leveling errors were negligible and the leveling offset was determined as 0.0 *ms* (1 *ms* = 10^-3^ *s*).

During the cruises, CTD casts were conducted near the CPIES stations to correct $\tau$. However, after adding the offsets determined from CTD casts, the estimates of the baroclinic velocity shears still differed from the velocity shear observed by the tall CM moorings varied by a large margin (Fig. S2). There are also large discrepancies in the mean velocities estimated across the section compared to those from the mooring observations and those derived from along-track altimeter observations; for example, the estimated mean surface velocity for RCM2 was negative (southwestward) with its value reaching 0.39 *m/s*. The significant surface countercurrent was not reported in previous studies, nor was it consistent with shipboard ADCP observations during the cruises (figures not shown). However, the mean surface geostrophic currents derived from altimeter in RCM1 were close to the ADCP measurements of the subsurface current (Fig. S2a). As the upper-layer velocities in RCM1 were highly barotropic, the temporal mean surface velocities derived from altimeters were validated by the *in situ* observations.

We consider that errors in the temporal mean velocities derived from altimeter were greatly reduced by the repeat along-track measurements. Thus, offsets of $\tau_{700}$ were determined using the velocity profiles from tall moorings and altimeter measurements taken during the observation period. However, the $\tau_{700}$ calculated from the CTD measurements were inappropriate to use to determine the offset. This could be attributed to two reasons: 1. Systematic errors arose from empirical functions for leveling. The errors can still exist even if the leveled $\tau_{700}$ snapshots were correct, and 2. CTD snapshots might have been contaminated by occasional events such as internal waves.

The procedures used to determine $\tau_{700}$ offsets for CPIES stations other than RES1 were as follows. Offsets from -2.0 *ms* to 2.0 *ms*, with a step of 0.1 *ms*, were added to the raw $\tau_{700}$ in RES2; this is how the temperature and salinity profiles in RES1 and RES2 were estimated. Velocity shears near RCM1 were calculated using the thermal-wind relationship. The velocity profile of the full water column was then obtained by adding the direct observations of RCM1 to a reference level of 700 *m*. The offset for RES2 (0.0 *ms*) was determined by identifying the estimate with the lowest difference between the root-mean-square error and the above velocity profile. Offsets for RES3 (-0.3 *ms*) and RES4 (-1.1 *ms*) were then determined by repeating these procedures. We noted that the current velocity shear based on the mooring observation (Fig.S2a) is still not quite consistent with GEM-based shear, so we tried to narrow down the area where historical CTD/ARGO data were collected, but results showed little improvements in GEM estimation. We speculate that CTD casts near RC core region were too few to offer the information related to high-mode velocity shear, thus only the first-mode velocity shears remained and others were smoothed out. Fortunately, we have multiple direct current measurements to supplement the missed velocity field. The details to better estimate the RC core structure is listed in Section 2.


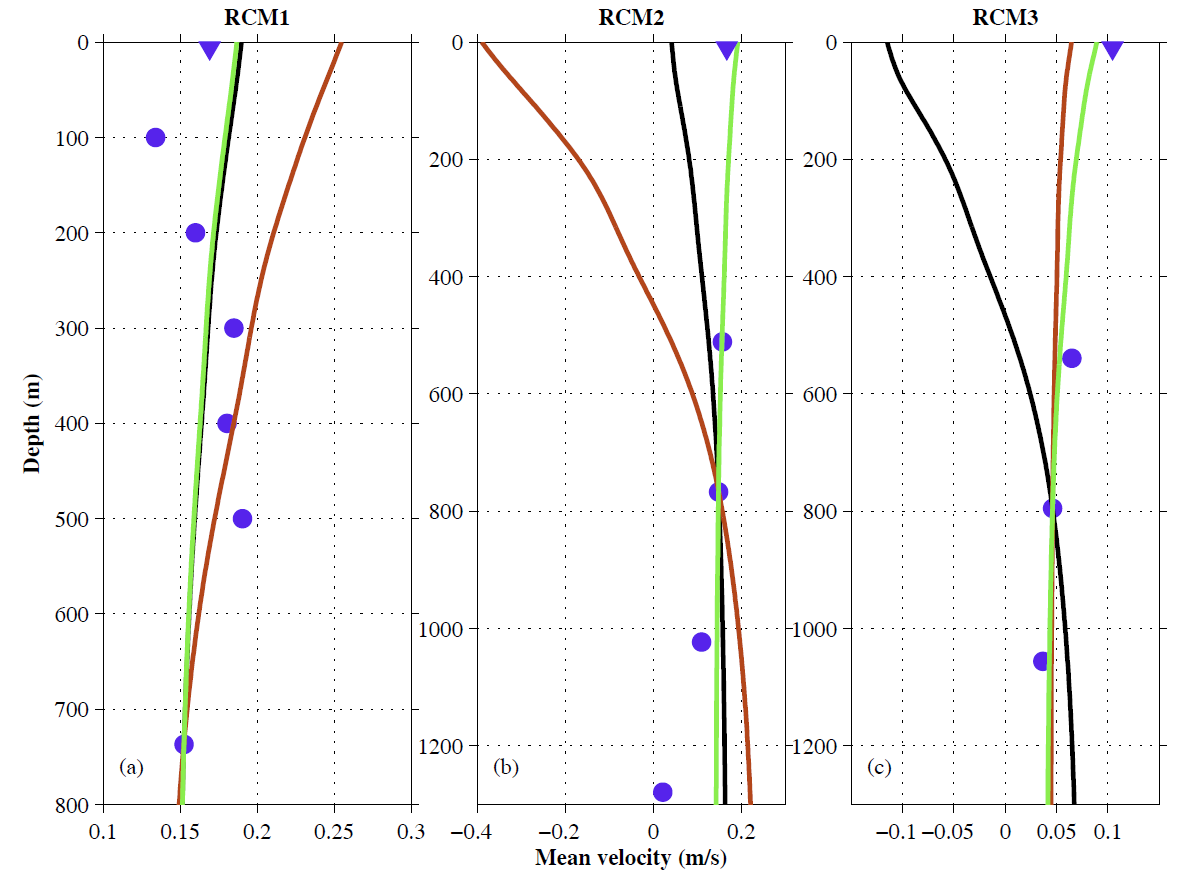


**Figure S2.** Estimated velocity profiles across the section for (a) RCM1, (b) RCM2, and (c) RCM3. Blue circles indicate observed temporal mean velocities across the section from tall moorings. Blue triangles indicate temporal mean surface velocities across the section derived from altimeter observations. Black, red, and green lines indicate the estimated velocity profiles without $\tau_{700}$ offsets, with $\tau_{700}$ offsets determined from CTD casts, and with $\tau_{700}$offsets determined from tall moorings and altimeter observations, respectively. The reference level for velocity estimations was 700 *m*.

**1.3 Optimal interpolation**

The optimal interpolation technique (Bretherton et al., 1976; Watts et al., 2001) was adopted to map corrected the $\tau_{700}$ values to fine horizontal grids with a resolution of 2 *km*. The scale of the correlation length was determined as 122 *km* from $\tau$ measurements. The baroclinic velocity field across the section was estimated with GEM based on the mapped $\tau_{700}$. The optimal interpolation method was not applied to map the deep velocity field from pressure data, as in previous studies (Watts et al., 2001; Andres et al., 2008a; Donohue et al., 2010), because there were too few CPIESs and because the bottom pressure records from RES1 were badly correlated with those of the other stations (Fig. S3b). Thus, total velocities (*V_bc_o_*_i_, where “*bc*” indicates “*baroclinic*” and “oi” indicates “optimal interpolation”) were estimated by a new and different method that is documented in the next section.


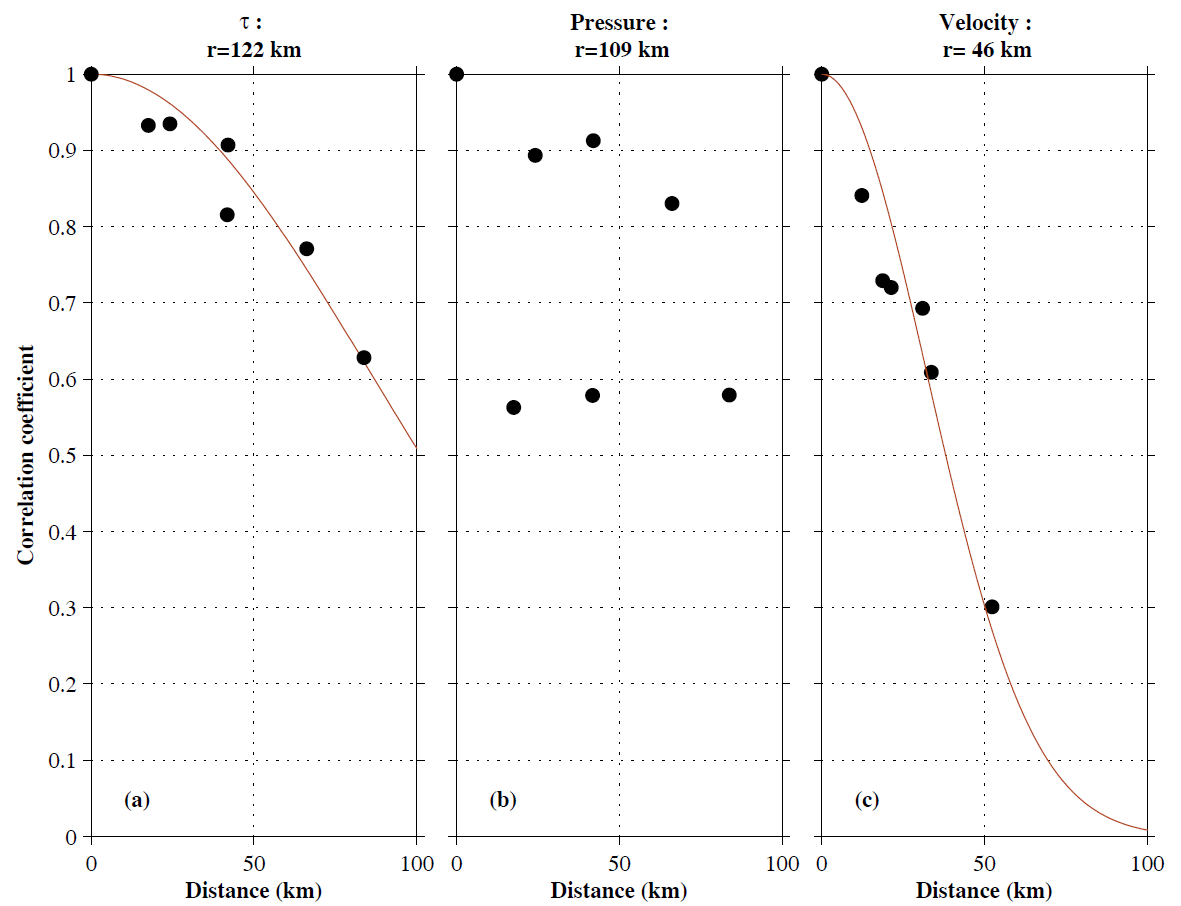


**Figure S3.** Fitted correlation function (red lines) for (a) $\tau_{700}$, (b) bottom pressure, and (c) leveled velocity at 500 *m*. Black dots indicate correlations measured every two stations.

**2. Estimating total velocity**

This section documents the procedures used to obtain absolute velocities from direct current observations and baroclinic velocity components, the latter had already been obtained using the methods outlined in the previous section. For a better accuracy, four estimates based on various reference depths (500 *m*, 700 *m*, 1000 *m*, and 1300 *m*) were made and the final estimate was obtained by designating depth-dependent weights for each estimation. The detailed procedure is as follows.

**2.1 Grouping observational records**

Tall mooring instruments were grouped by their nominal depths (Table S2) that were closest to each reference level. For each estimate, as many current records from CPIES as possible should be utilized because there are too few data points to use for further optimal interpolation. The exception was that RES2 was not utilized for estimates in the upper layers (Estimate No. 1, 2, and 3) because the velocities in RES2 were too small and there was a great velocity shear.

**Table S2** Instruments groupings and reference depths for each estimate. The missing RCM2 was not utilized for the second year estimation. “*d*” indicates the depth for the estimate.

| No. | Reference level (m) | Depth range(m) | Weights | Station | Instrument | Nominal depth (m) |
| --- | --- | --- | --- | --- | --- | --- |
| 1 | 500 | 0~500 | 1 | RCM1 | ADCP | 580 |
|  |  |  |  | RCM2 | CM | 511 |
|  |  |  |  | RCM3 | CM | 539 |
|  |  | 500~700 | $\frac{700-d}{700-500}$ | RES1 | CPIES | 732 |
|  |  |  |  | RES3 | CPIES | 1494 |
|  |  |  |  | RES4 | CPIES | 1721 |
| 2 | 700 | 500~700 | $\frac{d-700}{700-500}$ | RCM1 | CM | 737 |
|  |  |  |  | RCM2 | CM | 767 |
|  |  |  |  | RCM3 | CM | 795 |
|  |  | 700~1000 | $\frac{1000-d}{1000-700}$ | RES1 | CPIES | 732 |
|  |  |  |  | RES3 | CPIES | 1494 |
|  |  |  |  | RES4 | CPIES | 1721 |
| 3 | 1000 | 700~1000 | $\frac{1000-d}{1000-700}$ | RCM2 | CM | 1023 |
|  |  |  |  | RCM3 | CM | 1056 |
|  |  | 1000~1300 | $\frac{d-1000}{1300-1000}$ | RES3 | CPIES | 1494 |
|  |  |  |  | RES4 | CPIES | 1721 |
| 4 | 1300 | 1000~1300 | $\frac{1300-d}{1300-1000}$ | RCM2 | CM | 1279 |
|  |  |  |  | RCM3 | CM | 1056 |
|  |  |  |  | RES2 | CPIES | 1212 |
|  |  | >1300 | 1 | RES3 | CPIES | 1494 |
|  |  |  |  | RES4 | CPIES | 1721 |

- 1. **Estimating velocity based on reference depths**

For each estimate, the velocities of the reference depth were obtained by adding the baroclinic velocity shear estimated in section S1 to the grouped current measurements (Fig. S4). An optimal interpolation with a correlation length scale of 46 *km* (Fig. S3c) was then applied to these velocities and the *V_ref_oi_* value (where “*ref*” indicates the reference level) were obtained. The absolute velocities (*V_total_oi_*) are the sum of mapped barotropic velocities (*V_ref_oi_*) and previously estimated baroclinic velocity field (*V_bc_oi_*).


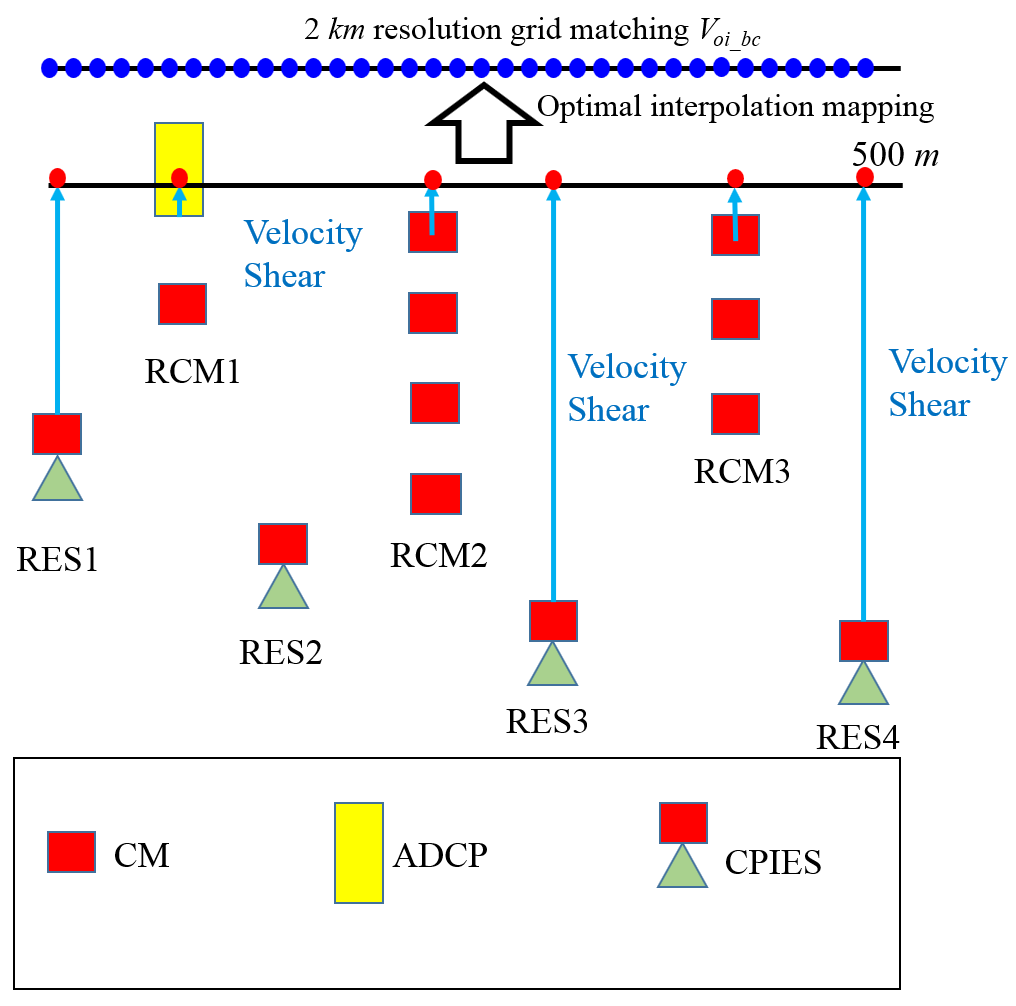


**Figure S4.** Schematic illustration of the velocity estimation based on a reference level of 500 *m* (Estimate No. 1). The rough positions of the mooring instruments are demonstrated. The baroclinic velocity shear (blue arrows) were obtained using the gravest empirical mode. Red dots indicate the positions at which the direct current observations were leveled. Leveled velocities were further mapped onto a 2 *km* resolution grid (blue dots) using optimal interpolation.

**2.3 Depth-weighted estimates**

Weights were given to each estimate, based on the depth of velocity we needed to calculate, because we had four sets of estimates although the final one used should be unique. For example, in the estimate of the velocities at 650 *m*, a larger weight ((650-500)/(700-500)=0.75) was given to the 2*nd* estimates, with a reference depth of 700 *m*, while a smaller one ((700-650)/(700-500)=0.25) was given to the 1*st* estimate with a reference depth of 500 *m*. The weights of the other estimates are zero.

The advantage of this technique (Fig. S5) is that the best use was made of the direct current observations from the mooring system. Moreover, the estimated velocities are quite consistent with observation records at the mooring positions; the estimated profiles are continuous and naturally smooth.


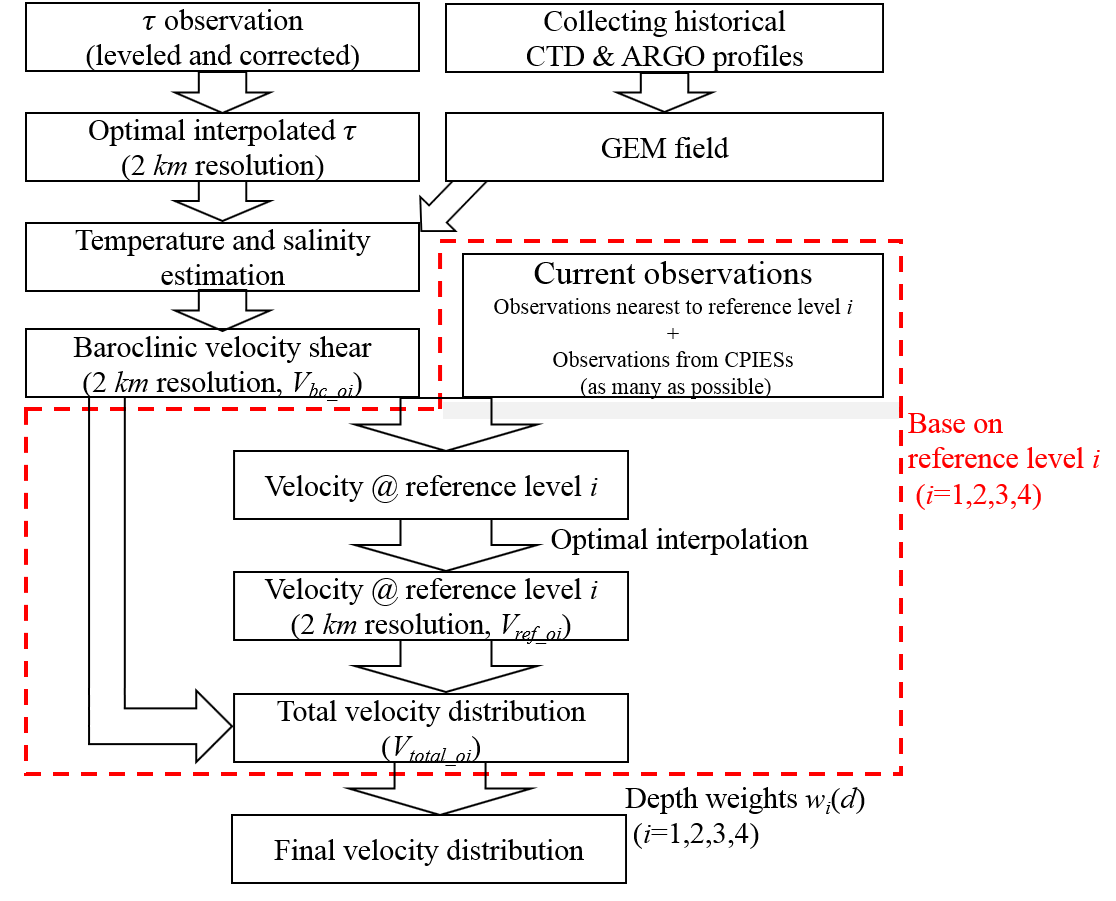
**Figure S5.** The flowchart for velocity estimation.
